# Cyanate Assimilation by the Alkaliphilic Cyanide-Degrading Bacterium *Pseudomonas pseudoalcaligenes* CECT5344: Mutational Analysis of the *cyn* Gene Cluster

**DOI:** 10.3390/ijms20123008

**Published:** 2019-06-20

**Authors:** Lara Paloma Sáez, Purificación Cabello, María Isabel Ibáñez, Víctor Manuel Luque-Almagro, María Dolores Roldán, Conrado Moreno-Vivián

**Affiliations:** 1 Departamento de Bioquímica y Biología Molecular, Edificio Severo Ochoa, 1ª Planta, Campus de Rabanales, Universidad de Córdoba, 14071 Córdoba, Spain; bb2samel@uco.es (L.P.S.); isaibga@gmail.com (M.I.I.); b42lualv@uco.es (V.M.L.-A.); bb2rorum@uco.es (M.D.R.); 2 Departamento de Botánica, Ecología y Fisiología Vegetal, Edificio Celestino Mutis, Campus de Rabanales, Universidad de Córdoba, 14071 Córdoba, Spain; bv1cahap@uco.es

**Keywords:** *Pseudomonas pseudoalcaligenes*, cyanate, cyanase, cyanide, jewelry residue, nitrate, nitrite

## Abstract

The alkaliphilic bacterium *Pseudomonas pseudoalcaligenes* CECT5344 can grow with cyanate, cyanide, or cyanide-containing industrial residues as the sole nitrogen source, but the assimilation of cyanide and cyanate takes place through independent pathways. Therefore, cyanide degradation involves a chemical reaction between cyanide and oxaloacetate to form a nitrile that is hydrolyzed to ammonium by the nitrilase NitC, whereas cyanate assimilation requires a cyanase that catalyzes cyanate decomposition to ammonium and carbon dioxide. The *P. pseudoalcaligenes* CECT5344 *cynFABDS* gene cluster codes for the putative transcriptional regulator CynF, the ABC-type cyanate transporter CynABD, and the cyanase CynS. In this study, transcriptional analysis revealed that the structural *cynABDS* genes constitute a single transcriptional unit, which was induced by cyanate and repressed by ammonium. Mutational characterization of the *cyn* genes indicated that CynF was essential for *cynABDS* gene expression and that nitrate/nitrite transporters may be involved in cyanate uptake, in addition to the CynABD transport system. Biodegradation of hazardous jewelry wastewater containing high amounts of cyanide and metals was achieved in a batch reactor operating at an alkaline pH after chemical treatment with hydrogen peroxide to oxidize cyanide to cyanate.

## 1. Introduction

Cyanide is a toxic chemical that inhibits different metalloproteins by its binding to the metal cofactors. Therefore, the inhibition of cytochrome *c* oxidase by cyanide blocks the aerobic respiratory electron transport chain, although many microorganisms tolerate cyanide because they present alternative cyanide-insensitive oxidases [1,2]. In addition, some of them are also able to degrade this toxic compound through different hydrolytic, oxidative, reductive, or substitution/addition pathways that generate ammonium, which is used as a nitrogen source [3,4,5,6,7,8]. Most cyanide-degrading microorganisms grow at a neutral pH, so a significant amount of cyanide may evaporate as HCN (*pK_a_* 9.2). However, the alkaliphilic bacterium *Pseudomonas pseudoalcaligenes* CECT5344 grows with free cyanide or metal–cyanide complexes as the sole nitrogen source under alkaline conditions (pH 9.5–10), avoiding cyanide volatilization [7,9,10]. In this bacterium, the cyanide degradation pathway requires a malate:quinone oxidoreductase that converts malate into oxaloacetate, which reacts chemically with cyanide, forming a cyanohydrin (hydroxynitrile) that is further hydrolyzed by the nitrilase NitC encoded by the *nit1C* gene cluster, generating ammonium [11,12]. 

Liquid waste containing cyanide and derivatives is generated at a large scale by different industrial activities, such as mining and metal processing, electroplating, coal coking, and nitrile polymer synthesis [5,8,13,14,15,16,17]. These cyanide-containing wastes often present heavy metals and metalloids, increasing their toxicity and becoming hazardous effluents that are difficult to remove from the environment due to the very high stability of the metal–cyanide complexes. The jewelry industry in Córdoba (Spain) produces several tons of alkaline residue (pH > 13) each year that contains up to 40 g/L cyanide (*ca*. 1.5 M), as free cyanide and both weak and strong metal–cyanide complexes [18]. Several physical and chemical treatments for cyanide removal have been described, including alkaline chlorination, SO_2_/air mixture (INCO process), copper-catalyzed hydrogen peroxide oxidation, ozonation, iron precipitation, and electrolytic decomposition. However, these methods are expensive, require special equipment and maintenance, and are usually inappropriate to degrade metal–cyanide complexes. Therefore, cyanide biodegradation may be an interesting alternative to these treatments [5,13,14,15,16,17,19]. In this context, the strain *P. pseudoalcaligenes* CECT5344 was able to grow in a batch reactor at pH 9.5 in the presence of sodium cyanide as the sole nitrogen source, removing cyanide at an optimal rate of 2.3 mg/L × A_600_ × h [10]. In addition, this strain was also able to degrade a jewelry wastewater containing both free cyanide and metal–cyanide complexes, with a cyanide consumption rate of about 1.9 mg/L × A_600_ × h [18].

In the environment, cyanate is produced by the oxidative treatment of cyanide-containing waste or as a result of spontaneous cyanide photooxidation, so that cyanate and cyanide are frequently found as co-contaminants [20]. Inside the cells, cyanate may be generated during urea and carbamoyl phosphate metabolism [21,22]. Cyanate is less toxic than cyanide, but causes ascorbate depletion and reacts with nucleophilic groups, promoting the carbamoylation of amino acids and proteins [23,24,25]. In addition, cyanate may chelate metal centers in some enzymes like carbonic anhydrase and superoxide dismutase [22]. Microorganisms assimilate cyanate as a nitrogen source through the enzyme cyanase, which catalyzes the decomposition of cyanate and bicarbonate into ammonium and carbon dioxide [22,25,26,27,28,29,30]. Moreover, some anammox bacteria use cyanate, resulting in nitrogen loss (cyanammox) [31], and ammonia-oxidizing archaea may also use cyanate as the energy source [32]. In this sense, a reciprocal feeding pattern has been proposed to explain how ammonia oxidizers without cyanase use cyanate as the sole source of energy, reductant, and nitrogen. Therefore, most nitrite oxidizers contain cyanase, and ammonium formed by this enzyme is released to the media and used by the associated ammonia oxidizers, which in turn generate the nitrite consumed by the nitrite oxidizers, allowing full nitrification from cyanate [32]. 

The crystal structure of the *Escherichia coli* cyanase reveals that the active enzyme is a homodecamer of 17 kDa subunits, which are organized into five identical dimers. Each monomer is composed of an N-terminal α-helix bundle and a C-terminal catalytic domain with a unique open fold, and the active site is located between pairs of dimers, including residues from four adjacent subunits [25]. A similar overall structure has been described for the cyanase from *Serratia proteamaculans* [30].

In bacteria, the cyanase c*ynS* gene is often clustered together with other genes involved in cyanate metabolism. Therefore, in *E. coli*, the *cyn* cluster includes three additional genes: *cynT*, *cynX*, and *cynR*, which code for a carbonic anhydrase, a putative cyanate transporter belonging to the major facilitator superfamily (MFS), and a positive regulator of the LysR family, respectively [33,34,35,36,37]. A similar operon including these four *cyn* genes was described for *Chromobacterium violaceum* [38]. Some cyanobacteria present a tricomponent ABC-type transporter encoded by the *cynABD* genes, which are located either immediately upstream from the cyanase *cynS* gene or separated by other putative genes, depending on the cyanobacterial strain [39,40]. In *Synechococcus elongatus*, the ABC-type cyanate transporter also shows a nitrite transport capacity. However, the main physiological role of this CynABD transporter is to allow efficient cyanate uptake because it shows a much higher affinity for cyanate than for nitrite, and low concentrations of cyanate competitively inhibit nitrite transport [41]. On the other hand, the transport of cyanate by a nitrate permease was also reported in *Azotobacter chroococcum* [42]. 

The alkaliphilic cyanide-degrading bacterium *P. pseudoalcaligenes* CECT5344 may use cyanate as the sole nitrogen source for growth, and its cyanase activity is induced by both cyanate and cyanide. However, cyanate is not an intermediate in the cyanide degradation pathway of this strain since a cyanase defective mutant (CynS^−^) showed a similar growth rate with cyanide as the sole nitrogen source to the wild-type strain [43]. The *cyn* gene cluster of *P. pseudoalcaligenes* CECT5344 includes the structural genes *cynABDS* encoding the three components of a putative ABC-type cyanate transporter and the cyanase, respectively. An additional gene (*cynF*) that codes for a putative σ^54^-dependent transcriptional regulator is located 268-bp upstream from *cynA*, in the opposite direction to *cynABDS* [43]. The whole genome of *P. pseudoalcaligenes* CECT5344 has recently been sequenced and the *cynFABDS* genes were annotated with the accession numbers BN5_0438 to BN5_0442, respectively [44,45,46]. The cyanase of *P. pseudoalcaligenes* CECT5344 has been previously purified and characterized. The purified enzyme, which showed an apparent molecular mass of 16 kDa, an optimum pH of 8.5, and an optimum temperature of 65 °C, was also used to design a cyanate biosensor for bioremediation processes [43,47]. 

In this work, we have carried out a mutational analysis of the *cyn* gene cluster of the alkaliphilic bacterium *P. pseudoalcaligenes* CECT5344, which revealed that the transcriptional regulator CynF is essential for expression of the structural *cynABDS* genes, and that in the absence of a functional CynABD transporter, cyanate can be incorporated into the cells by other alternative systems like nitrate/nitrite transporters. The regulation of cyanate assimilation was also studied in this bacterium. In addition, the degradation of a cyanide-containing jewelry industry wastewater, which was pre-treated with hydrogen peroxide to provoke cyanide oxidation to cyanate, was carried out in a batch reactor.

## 2. Results and Discussion

### 2.1. The cynFABDS Gene Cluster of P. pseudoalcaligenes CECT5344

**Figure 1:**
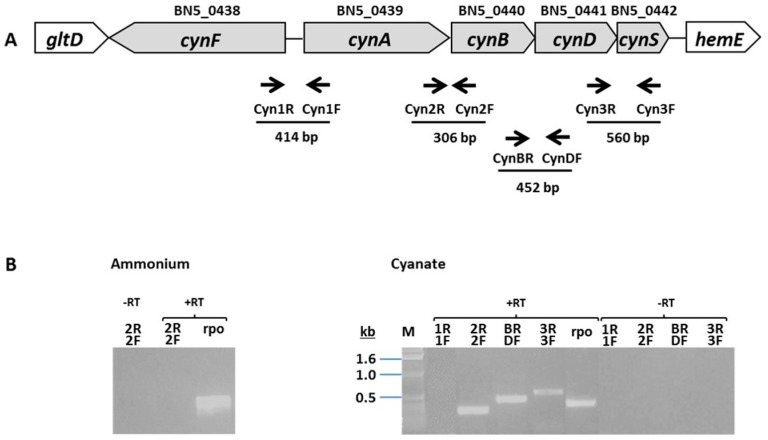
Gene arrangement (**A**) and reverse transcription polymerase chain reaction (RT-PCR) transcriptional analysis (**B**) of the *P. pseudoalcaligenes* CECT5344 *cyn* gene cluster. Primer pairs used for the amplification reactions (Table S2, Supplementary Materials) are shown, but not drawn to scale. The expected sizes of the PCR products are also given. Isolation of mRNA from ammonium- or cyanate-grown cells and PCR reactions were carried out as described in Materials and Methods, with (+RT) or without (–RT) reverse transcriptase. The housekeeping *rpoB* gene was used as the control.

The alkaliphilic cyanide-degrading bacterium *P. pseudoalcaligenes* CECT5344 harbors a *cyn* gene cluster (Figure 1A) that includes the *cynF* gene encoding a putative transcriptional regulator of the Fis family and the structural *cynABDS* genes, which are transcribed in the opposite direction to *cynF* and code for the periplasmic component, the inner membrane subunit and the cytosolic ATPase protein of a putative ABC-type cyanate transporter, and the enzyme cyanase (cyanate hydratase), respectively [43]. These genes were annotated in the whole genome sequence of *P. pseudoalcaligenes* CECT5344 with the accession numbers BN5_0438 to BN5_0442, respectively [44,45,46]. The genes located upstream from *cynF* encode the large and small glutamate synthase subunits, whereas genes downstream from *cynS* code for the uroporphyrinogen decarboxilase HemE and the insertion sequence element IS4 transposase. A putative σ^54^-dependent promoter (consensus RpoN or NtrA binding sequence) was identified upstream from the *cynA* gene, whereas a putative σ^70^ factor binding sequence (-10 and -35 boxes) was present upstream from the *cynF* gene (Figure S1, Supplementary Materials).

A thorough search of the genome databases reveals that the *cynFABDS* gene arrangement of *P. pseudoalcaligenes* CECT5344 is conserved in *Pseudomonas mendocina*, with all genes sharing 97–98% identity with those of the strain CECT5344. A similar gene distribution was also found in other species of *Pseudomonas* and different alpha, beta, and gamma proteobacteria, although showing less identity levels, suggesting that the cyanate assimilation capability is widely distributed among bacteria. However, only a few bacterial species also contain the *cioAB* genes encoding the cyanide-insensitive alternative oxidase that confers cyanide resistance in the strain CECT5344 [48]. Therefore, considering that *P. pseudoalcaligenes* CECT5344 possesses the *cyn* genes required for cyanate assimilation, the *cioAB* genes involved in cyanide tolerance, and the *nitC1* gene cluster needed for the assimilation of cyanide and nitriles, this strain is a good candidate to be used in the detoxification of industrial wastewaters containing both cyanide and cyanate as co-contaminants.

To determine if the structural *cynABDS* genes, which are divergently orientated to the regulatory *cynF* gene, are organized in a transcriptional unit, a reverse transcription polymerase chain reaction (RT-PCR) analysis was carried out using mRNA isolated from cells grown with ammonium or 2 mM cyanate and the indicated primer pairs (Figure 1). As the positive control, a 372 bp fragment of the constitutive *rpoB* gene coding for the β subunit of the RNA polymerase was amplified. Using mRNA from ammonium-grown cells, no amplification was observed (Figure 1B, left). However, when the mRNA from cells grown with cyanate was used for the RT-PCR reactions, fragments with the estimated sizes, expanding from the 3′-end of *cynA* to the 5′-end of *cynB*, from the 3′-end of *cynB* to the 5′-end of *cynD*, and from the 3′-end of *cynD* to the 5′-end of *cynS*, were successfully amplified (Figure 1B, right). As expected, no amplification was observed in the RT-PCR reaction using the primers corresponding to the 5′-ends of the *cynF* and *cynA* genes, which are divergently transcribed (Figure 1B, right). This result indicates that the structural *cynABDS* genes constitute a single transcriptional unit that is expressed in the presence of cyanate, but not when the cells use ammonium as the nitrogen source. This result agrees with the presence of a σ^54^ factor (NtrA) binding site in the *cynA* promoter (Figure S1, Supplementary Materials).

### 2.2. Regulation of the Cyanate Assimilation in P. pseudoalcaligenes CECT5344

*P. pseudoalcaligenes* CECT5344 can grow with cyanate as the sole nitrogen source at concentrations as high as 100 mM. Therefore, after 48 h growth, the cultures reached an absorbance at 600 nm (A_600_) of about 1.0 with100 mM cyanate, but only an A_600_ of about 0.27 with 200 mM cyanate. This elevated tolerance of the strain CECT5344 to cyanate, which is much higher than in other bacteria like *E. coli*, which only tolerates up to 20 mM cyanate [28], is of great interest for biodegradation and bioremediation applications.

To understand the regulation of the cyanate assimilation process, the cyanase enzymatic activity was determined in *P. pseudoalcaligenes* CECT5344 cells after 8 hours culture in the presence of different nitrogen sources. In the wild-type strain CECT5344, the cyanase was significantly induced by cyanate and strongly repressed by ammonium, whereas a basal level of cyanase activity was found in the absence of a nitrogen source (nitrogen starvation) or with other nitrogen sources like nitrate or glutamate (Table 1). This regulatory pattern is typically observed in processes involved in the assimilation of alternative nitrogen sources, like nitrate assimilation, which are usually induced by the substrate and inhibited or repressed by the product. For this reason, the highest nitrate reductase activity in *P. pseudoalcaligenes* CECT5344 wild-type cells was observed in the presence of nitrate, whereas the lowest level of activity was found in media with ammonium (Table 1). Induction of the *cynABDS* genes by cyanide and a cyanide-containing jewelry residue was also observed in a DNA microarray analysis carried out in *P. pseudoalcaligenes* CECT5344 [49], and a quantitative proteomic analysis also revealed that the CynABDS proteins are present in cells grown in the jewelry wastewater at significantly higher levels than in ammonium-grown cells [18].

### 2.3. Mutational Analysis of the cyn Gene Cluster of P. pseudoalcaligenes CECT5344

In a previous work, it was reported that a cyanase-defective mutant (CynS^−^) of *P. pseudoalcaligenes* CECT5344 did not grow with cyanate as the sole nitrogen source, but was still able to grow with cyanide [43]. This result indicates that cyanase, an essential enzyme for cyanate assimilation, is not involved in cyanide degradation. Instead, this process requires the nitrilase NitC that hydrolyzes the cyanohydrin (hydroxynitrile) formed by a chemical reaction between cyanide and the oxaloacetate generated through a malate:quinone oxidoreductase associated with the cyanide-insensitive alternative oxidase CioAB [11,12]. To further characterize the assimilation of cyanate in the strain CECT5344 and to analyze the functional role of the *cyn* genes in this process, a mutant in the putative regulatory *cynF* gene (CynF^−^) and mutants lacking the predicted integral membrane and ATPase components of the ABC-type cyanate transporter, either without (CynBD^−^) or with a polar effect on the *cynS* gene (CynBDS^−^), have been constructed.

**Figure 2:**
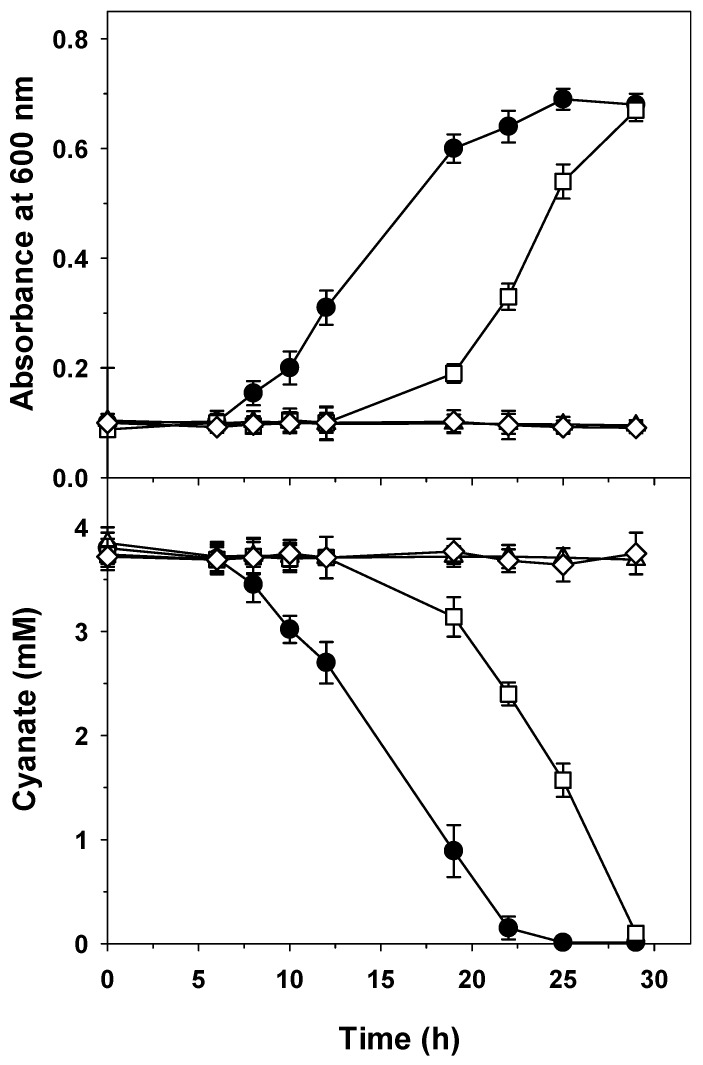
Growth and cyanate consumption of the wild-type strain (●) and the CynF^−^ (△), CynBD^−^ (☐), and CynS^−^ (◇) mutants of *P. pseudoalcaligenes* CECT5344 in media with cyanate. Cells were grown in the minimal medium M9 with 4 mM cyanate as the sole nitrogen source, and at the indicated times, the bacterial growth (A_600_) and cyanate concentration in the media were determined. Error bars show standard deviations of three independent replicates.

The mutational approach revealed that *cynF* and *cynS* genes were essential for cyanate assimilation in *P. pseudoalcaligenes* CECT5344 because both CynF^−^ and CynS^−^ mutant strains were unable to grow with 4 mM cyanate as the sole nitrogen source, and consequently, the consumption of cyanate was not detected (Figure 2). This result indicates that the Fis-like σ^54^-dependent transcriptional regulator CynF is required to induce the structural *cynABDS* genes and confirms that the cyanase CynS is essential for cyanate assimilation. The CynF^−^ mutant did not show cyanase activity in any of the nitrogen sources assayed (Table 1), confirming that the transcriptional regulator CynF was required to induce the cyanase *cynS* gene. In addition, the absence of cyanase activity in the wild-type strain in media with ammonium (Table 1) also indicates that the regulatory function of CynF is dependent on the general ammonium control, probably through the Ntr system and the σ^54^ factor, as suggested by the presence of an NtrA (RpoN) binding site upstream from the *cynA* gene (Figure S1, Supplementary Materials). Interestingly, the mutation in the CynF regulator also affected the nitrate reductase activity of *P. pseudoalcaligenes* CECT5344, which became constitutive and was neither subjected to nitrate induction nor ammonium repression, as occurred in the wild-type strain (Table 1). This suggests a possible interconnection between cyanate and nitrate assimilation processes that will be further investigated in the future. 

**Figure 3:**
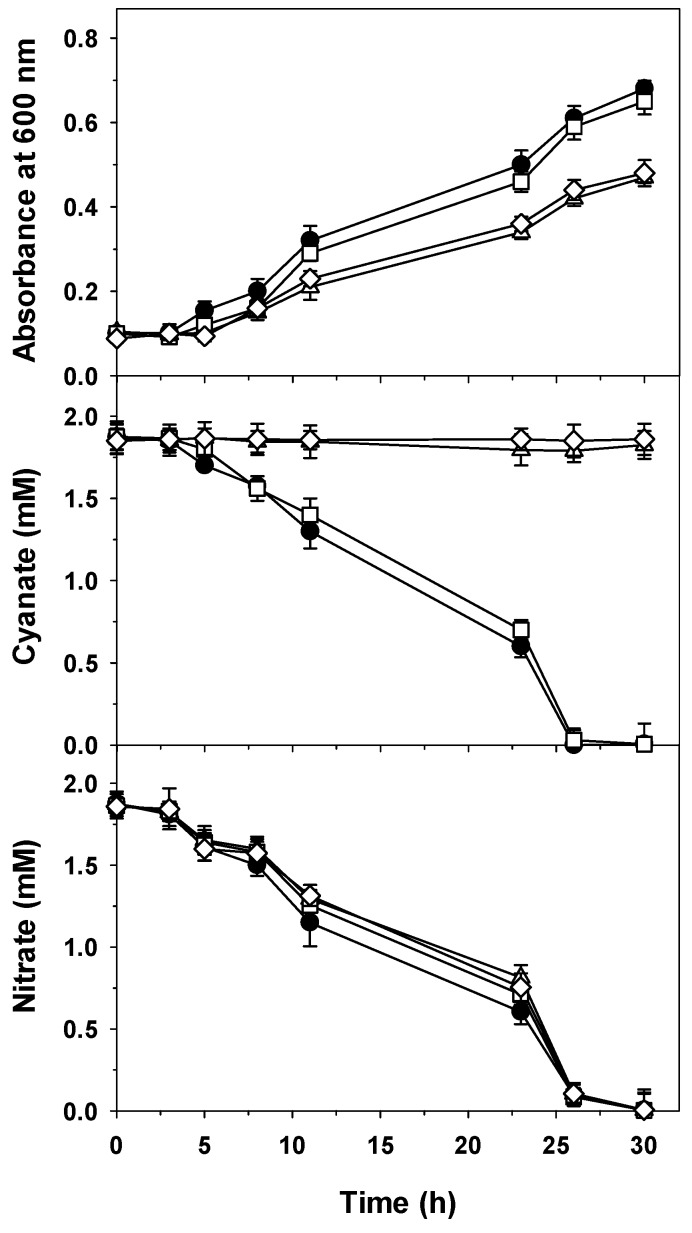
Growth, and cyanate and nitrate consumption of the wild-type strain (●) and the CynF^−^ (△), CynBD^−^ (☐), and CynS^−^ (◇) mutants of *P. pseudoalcaligenes* CECT5344 in media with cyanate and nitrate. Cells were grown in the minimal medium M9 with 2 mM cyanate and 2 mM nitrate as nitrogen sources, and at the indicated times, the bacterial growth (A_600_) and cyanate and nitrate concentrations were determined. Error bars show standard deviations of three independent replicates.

The non-polar CynBD^−^ mutant strain was able to grow using cyanate as the sole nitrogen source, but both bacterial growth and cyanate consumption were significantly delayed compared to the wild-type strain (Figure 2). This non-polar mutant expressed the *cynS* gene, as revealed by qRT-PCR (not shown). This result suggests the presence of another transport system that, rather than by cyanate, could be induced in the nitrogen starvation condition, in which the CynBD^−^ mutant is probably found when cyanate cannot be incorporated into the cells by the CynABD transporter. When the wild-type and mutant strains of *P. pseudoalcaligenes* CECT5344 were cultured with 2 mM cyanate plus 2 mM nitrate as simultaneous nitrogen sources, all strains were able to grow by consuming nitrate at similar rates, but interestingly, the non-polar CynBD^−^ mutant was also able to consume cyanate without delay and at a similar rate to the wild-type strain (Figure 3), suggesting that in the absence of a functional CynABD transporter, cyanate may be incorporated into the cells by an alternative transport system that is efficiently induced by nitrate. Therefore, an additional transport system that is induced by a nitrate or nitrogen deficiency condition may be responsible for cyanate transport in the CynBD^−^ mutant (Figure 2 and Figure 3). 

**Figure 4:**
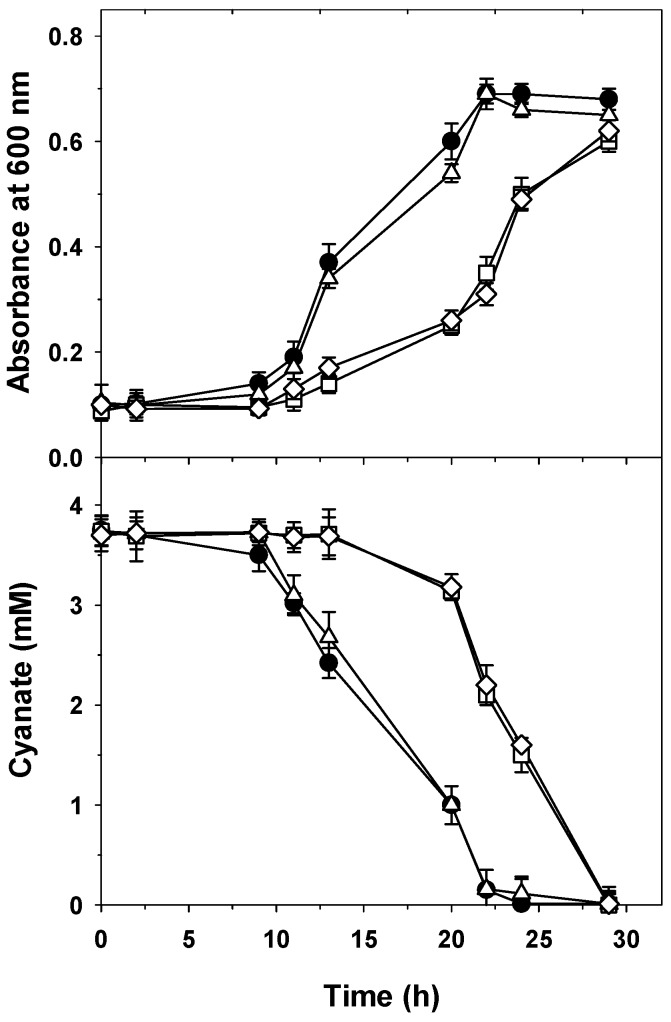
Growth and cyanate consumption in media with cyanate of the *P. pseudoalcaligenes* CECT5344 wild-type (●) and the single or double mutant strains CynX^−^ (△), CynBD^−^ (☐), and CynX^−^/CynBD^−^ (◇). Cells were grown in the minimal medium M9 with 4 mM cyanate as the sole nitrogen source, and at the indicated times, the bacterial growth (A_600_) and cyanate concentration in the media were determined. Error bars show standard deviations of three independent replicates.

The genome of *P. pseudoalcaligenes* CECT5344 contains a gene (BN5_3113), which was named *cynX* because it codes for a putative MFS-type transporter that shows about 30% identity with the *E. coli* CynX cyanate transporter. However, this gene is not closely located to other genes involved in the assimilation of cyanate, cyanide, nitrate, or other alternative nitrogen sources [45,46]. A recent DNA microarray transcriptomic analysis of the CECT5344 strain revealed that the *cynX* gene is expressed in a nitrogen starvation condition, but is not induced in response to sodium cyanide or a cyanide-containing jewelry residue [49]. To test if the putative CynX protein mediates the delayed cyanate transport observed in the *P. pseudoalcaligenes* CynBD^−^ mutant (Figure 2), single CynX^−^ and double CynX^−^/CynBD^−^ mutant strains were also constructed. In media with cyanate as the sole nitrogen source, growth and cyanate uptake were similar in the CynX^−^ mutant and the wild-type strain (Figure 4), whereas the double CynX^−^/CynBD^−^ mutant displayed a significant delay in both growth and cyanate consumption, as observed in the single CynBD^−^ mutant (Figure 2 and Figure 4). However, in the presence of cyanate and nitrate, all these strains were able to grow at similar rates by consuming both cyanate and nitrate. These results suggest that the *cynX* gene of *P. pseudoalcaligenes* is not involved in the transport of cyanate, and instead, a putative nitrate/nitrite transporter could mediate the delayed cyanate consumption observed in both single CynBD^−^ and double CynX^−^/CynBD^−^ mutant strains. In this regard, the uptake of cyanate by a nitrate permease has been reported in *Azotobacter chroococcum* [42], and the CynABD cyanate transporter of *Synechococcus elongatus* is also able to transport nitrite [41], so that a certain degree of crossed specificity may occur between some bacterial cyanate and nitrate/nitrite transport systems. Sequencing of the *P. pseudoalcaligenes* CECT5344 genome has revealed the presence of different putative nitrate/nitrite transporters of both ABC- and MFS-type [44,45,46]. Therefore, the nitrate assimilation *nas* gene cluster of the strain CECT5344 includes the *nasFED* genes (BN5_2114 to BN5_2116) encoding a tricomponent ABC-type nitrate transporter and the *narT* gene (BN5_2121) that codes for a putative MFS-type nitrate/nitrite transporter, in addition to the *nirBD* and *nasA* genes (BN5_2123 to BN5_2125) encoding the large and small subunits of the NADH-dependent nitrite reductase and the nitrate reductase catalytic subunit, respectively [44]. These *nas* genes are induced by nitrate and are also expressed under nitrogen deficiency [49]. In addition, some proteins encoded by this *P. pseudoalcaligenes nas* gene cluster were found to be induced by cyanide-containing jewelry wastewater in a recent quantitative proteomic analysis [18]. 

### 2.4. Biodegradation of Jewelry Wastewater Treated with Hydrogen Peroxide to Oxidize Cyanide to Cyanate

As mentioned above, the tolerance to cyanate in the strain *P. pseudoalcaligenes* CECT5344 (up to 100 mM) is much higher than in other bacteria [28]. This fact may be of great interest for the biodegradation and bioremediation of industrial cyanide-containing residues, considering that cyanide can be oxidized to cyanate by physical–chemical treatments, thus reducing the toxicity of these residues and probably increasing the capacity of the bacteria to remove large amounts of these hazardous compounds. 

To investigate if a hydrogen peroxide pre-treatment of an industrial residue containing cyanide and metals can be used to oxidize cyanide to cyanate, jewelry wastewater with 4 mM free cyanide was treated with hydrogen peroxide with or without simultaneous ultraviolet (UV) irradiation. After 30 min treatment with 6 mM hydrogen peroxide, about 1.4 mM cyanate was detected, indicating that 35% of cyanide was converted into cyanate. An almost identical result was obtained when the residue was treated simultaneously with hydrogen peroxide and UV radiation (37% cyanide oxidation to cyanate), but no significant oxidation of cyanide to cyanate was observed when the residue was only irradiated with UV (not shown), indicating that UV irradiation was not effective for oxidizing the cyanide present in the industrial residue under our experimental conditions. When this pre-treatment was performed using 10 mM hydrogen peroxide, about 80% of the cyanide present in the jewelry residue was oxidized to cyanate (3.2 mM cyanate was detected after 30 min treatment). In addition, we also tested the tolerance of *P. pseudoalcaligenes* CECT5344 to hydrogen peroxide in media with acetate and ammonium as carbon and nitrogen sources, respectively. This strain was able to tolerate up to 10 mM hydrogen peroxide without affecting its growth, although 25 mM hydrogen peroxide caused a significant inhibition of bacterial growth (not shown).

**Figure 5:**
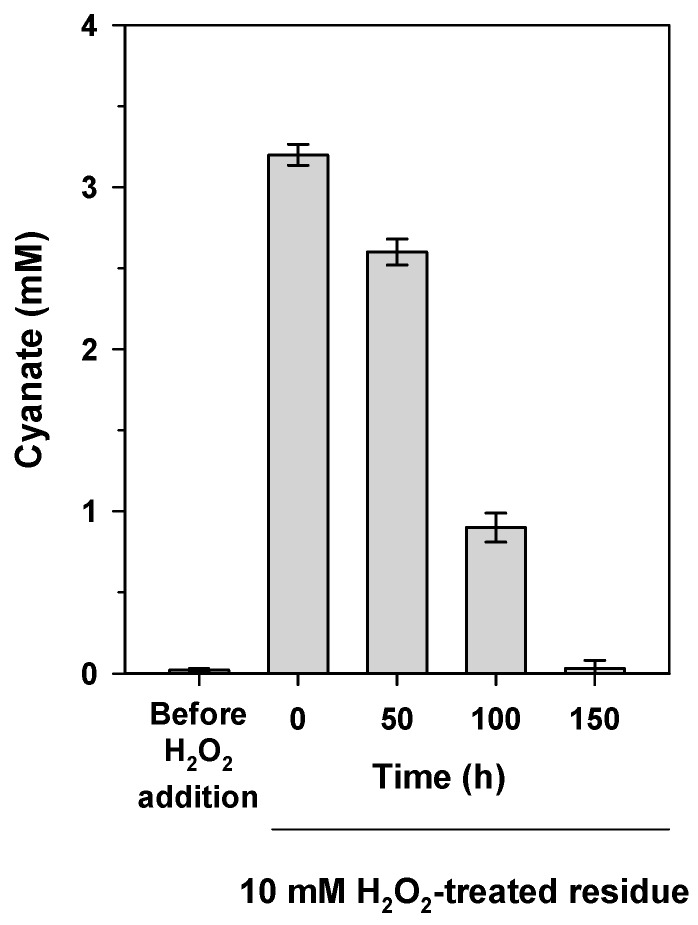
Biodegradation in a batch reactor of jewelry industry wastewater pre-treated with hydrogen peroxide. Before inoculation with *P. pseudoalcaligenes* CECT5344, a jewelry residue containing metals and 4 mM free cyanide was treated in the reactor with 10 mM hydrogen peroxide to oxidize cyanide to cyanate. The concentration of cyanate was determined before and after the treatment, at the indicated times during the experiment. Error bars show standard deviations of three independent replicates.

Biodegradation of the jewelry wastewater containing 4 mM free cyanide pre-treated with 10 mM hydrogen peroxide was successfully carried out in a batch reactor operating at a constant pH of 9.5 to prevent cyanide volatilization. The treatment with hydrogen peroxide generated about 3.2 mM cyanate, which was totally consumed by *P. pseudoalcaligenes* CECT5344 after 150 h (Figure 5). No significant cyanide concentrations were detected during the experiment. It is worth noting that this jewelry residue also contains different metals like copper, iron, and zinc [18]. This result confirms that the chemical oxidation of cyanide to cyanate may facilitate degradation in bioreactors of industrial residues containing cyanide and metal–cyanide complexes.

## 3. Materials and Methods

### 3.1. Chemicals

Potassium cyanate, ammonium chloride, potassium nitrate, sodium glutamate, and sodium acetate were supplied by Sigma–Aldrich (St. Louis, MO, USA). All other chemicals used in the study were of analytical grade. Solutions were prepared in water that was previously passed through a Milli-Q system from Millipore (Bedford, MA, USA). The jewelry industry wastewater containing cyanide and metals was supplied by the companies Avenir S.L. and Gemasur S.L. (Córdoba, Spain). Waste containing cyanide or other toxic compounds was disposed by the Environmental Protection Unit, University of Córdoba. 

### 3.2. Bacterial Strains, Plasmids, and Growth Conditions

The bacterial strains and plasmids used in this study are listed in Table S1 (Supplementary Materials). *P. pseudoalcaligenes* strains were grown in Erlenmeyer flasks filled to 16% of their total volume with M9 minimal medium [50], and the pH was adjusted to 9.0 or 9.5 with 0.1 N sodium hydroxide. Sodium acetate (50 mM) was used as the carbon source and the different nitrogen sources (ammonium chloride, potassium cyanate, potassium nitrate, and sodium glutamate) were used at the indicated concentrations. The jewelry residue was added to the culture media after dilution to obtain a free cyanide concentration of 4 mM (about 6 mM total cyanide due to the presence of metal–cyanide complexes). *Escherichia coli* strains were grown in test tubes with metal caps, filled with 3 mL LB medium. For solid media preparation, 1.5% bacteriological agar was added. Cells were incubated at 30 °C (*P. pseudoalcaligenes*) or 37 °C (*E. coli*) on a rotatory shaker at 230 rpm. Then, required media were supplemented with ampicillin (100 μg/mL), kanamycin (25 μg/mL), nalidixic acid (10 μg/mL), or gentamicin (20 μg/mL). 

Experiments in the bioreactor were conducted in a Biostat^®^ C plus (Sartorius BBI systems) 10 L reactor using the following operational procedure. The reactor was loaded with M9 minimal medium [50] containing 50 mM sodium acetate as the carbon source, and the jewelry wastewater was added at a final free cyanide concentration of 4 mM (about 6 mM total cyanide). Before inoculation, pre-treatment with 10 mM hydrogen peroxide was carried out as described below. To begin the biodegradation process, an appropriate volume of inoculum from a *P. pseudoalcaligenes* CECT5344 overnight culture in M9 medium with ammonium as the nitrogen source was added to obtain an A_600_ of approximately 0.3. The temperature was kept at 30 °C and the pH was adjusted to 9.5 with NaOH, and was then further controlled to remain at this value. Agitation (450 rpm) and dissolved oxygen saturation (10%) were also maintained. To prevent possible HCN losses during the experiments, the bioreactor exhaust cooler was connected to a washing flask containing a concentrated NaOH solution.

### 3.3. Analytical Determinations

Bacterial growth was monitored by following the absorbance of the culture at 600 nm (A_600_). Nitrate, nitrite, and ammonium concentrations were determined as previously described [9,43,51]. The concentration of free cyanide was determined colorimetrically [52] and the cyanate concentration was quantified by its chemical conversion into ammonium as described hitherto [43]. The protein concentration was assayed by a modified version of the Lowry procedure [53]. 

### 3.4. Enzyme Activity Assays

Cells were harvested and broken by sonication (3 pulses of 5 s at 90 W). Crude extracts were centrifuged and supernatants corresponding to the subcellular soluble fractions were used as a source to assay enzyme activity. Nitrate reductase activity was measured with methyl viologen reduced in the presence of dithionite, and by quantifying nitrite production [51]. Cyanase was determined following the method of Anderson [26], as previously described [43]. One unit of activity is defined as the amount of enzyme producing one micromol of product per minute under the assay conditions.

### 3.5. Peroxidation of the Cyanide-Containing Industrial Wastewater 

Cyanide present in the jewelry residue was oxidized to cyanate by chemical treatment with hydrogen peroxide. The residue, diluted in M9 medium to obtain a final concentration of 4 mM free cyanide, was treated with 6–10 mM H_2_O_2_ for 15 min, with or without UV irradiation using a 20 W UV lamp (Philips, Eindhoven, The Netherlands). After treatment, cyanate and free cyanide were determined as indicated above. 

### 3.6. DNA Manipulations, Sequencing, and Generation of Mutant Strains

DNA manipulations were performed according to the methods described by Sambrook and Russel [50]. Sequencing of DNA fragments was carried out using the facilities of the Genomic Unit of the Central Service for Research Support (SCAI) at the University of Córdoba. The *P. pseudoalcaligenes* CECT5344 whole genome sequence is available on the EMBL database under the Accession No. LK391695 [44,45,46].

The construction of a cyanase-defective mutant strain (CynS^−^) of *P. pseudoalcaligenes* CECT5344 by the insertion of a gentamicin resistance cassette into a central region of the *cynS* gene (*cynS::Gm*) was previously described [43]. To disrupt the *cynF* gene, a 2.1 kb DNA fragment containing this gene was amplified by PCR using genomic DNA and the primers CynLF10 and CynLR7 (Table S2, Supplementary Materials). The PCR product was cloned into pGEM-T Easy, resulting in the plasmid pGEMT-*cynF*, and further subcloned into the pBmod vector using *Eco*RI restriction sites, yielding the pBmod-*cynF* plasmid. The pBmod vector was constructed by removing the *Sal*I site between the *Eco*RV and *Hinc*II sites in the ampicillin-resistant pBluescript plasmid. A kanamycin resistance cassette was inserted into the *Sal*I site present in the central region of the *cynF* gene, generating the pBmod-*cynF::Km* plasmid. Finally, the *cynF* gene with the insertion of the kanamycin cassette was subcloned into the mobilizable plasmid pK18mob to generate the construct pK18mob-*cynF::Km*. This final plasmid was used for conjugational mating between *E. coli* S17-1 (donor strain) and *P. pseudoalcaligenes* CECT5344 (nalidixic acid-resistant receptor strain) to obtain the CynF^−^ mutant by homologous recombination, which was selected in media with nalidixic acid and kanamycin. The authenticity of the insertion was confirmed by PCR with the primers CynLF10/CynLR7 and by DNA sequencing. To disrupt the *cynBD* genes, both genes were amplified by PCR reactions using genomic DNA and the primer pairs CynLR9/CynLF11 to amplify *cynB* and CynLR8/CynLF8 to amplify *cynD* (Table S2, Supplementary Materials). These PCR products were cloned into pGEM-T Easy (ampicillin resistant) and pK18mob (kanamycin resistant), resulting in the plasmids pGEMT-*cynB* and pK18mob-*cynD*, respectively. Then, the *Bam*HI-*Hin*dIII fragment from pGEMT-*cynB* containing the *cynB* gene was subcloned into pK18mob-*cynD*, creating the pK18mob-*cynBD* plasmid. In this construct, a gentamicin cassette was inserted using the *Bam*HI restriction site present between both *cynB* and *cynD* genes. Two resulting plasmids pK18mob-*cynBD::Gm* were generated, depending on the antibiotic resistance cassette orientation (the same or opposite direction with respect to the *cyn* genes). Each plasmid was transferred by conjugational mating from the donor strain *E. coli* S17-1 to the nalidixic acid-resistant *P. pseudoalcaligenes* CECT5344 receptor, resulting in mutants without or with a polar effect on the *cynS* gene located downstream (CynBD^−^ or CynBDS^−^ mutants, respectively). Transconjugants obtained by homologous recombination were selected in media with selective antibiotics and the authenticity of the mutants was confirmed by a PCR reaction and DNA sequencing.

To generate the single CynX^−^ and the double CynX^−^/CynBD^−^ mutant strains, an internal 533 bp DNA fragment of the *cynX* gene was amplified by PCR using the primers 3113FB and 3113RH (Table S2, Supplementary Materials). This PCR product was cloned into the *Bam*HI and *Hin*dIII sites of the pK18mob plasmid to obtain the pK18mob-*cynX* kanamycin-resistant construct, which was mobilized by conjugational mating from *E. coli* S17-1 to the nalidixic acid-resistant *P. pseudoalcaligenes* CECT5344 wild-type strain (to generate the single CynX^−^ mutant) or to the gentamicin-resistant CynBD^−^ mutant strain (to generate the double CynX^−^/CynBD^−^ mutant). The authenticity of these mutants was confirmed by PCR reactions and DNA sequencing.

### 3.7. RT-PCR Transcriptional Analysis of the cyn Gene Cluster 

*P. pseudoalcaligenes* CECT5344 was cultured with 2 mM cyanate or 2 mM ammonium, and when about 50% of the nitrogen source was consumed, cells were harvested and washed in the TEG buffer, which contains 25 mM Tris-HCl (pH 8.0), 10 mM EDTA, and 1% glucose. RNA isolations, DNase incubations, the synthesis of total cDNA, RT-PCR, and qRT-PCR reactions were carried out as previously described [54]. A negative control without retrotranscriptase was made for each sample. The specific oligonucleotides used for PCR reactions are listed in Table S2 (Supplementary Materials) and shown in Figure 1A.

## 4. Conclusions

The *cynFABDS* gene cluster of the alkaliphilic cyanide-degrading bacterium *P. pseudoalcaligenes* CECT5344 is required for cyanate assimilation, but not for cyanide degradation. Mutational analysis reveals that the product of the *cynF* gene is essential for the expression of the structural *cynABDS* genes, which constitute a single transcriptional unit that is induced by cyanate and repressed by ammonium. Cyanate transport takes place by the cyanate-induced, ABC-type CynABD system, but nitrate/nitrite transporters may be used for cyanate uptake because mutants lacking the *cynBD* genes incorporate cyanate in the presence of both cyanate and nitrate. The mutation of a putative *cynX* gene that is not clustered together with the *cynFABD* genes reveals that this gene is not involved in cyanate transport. *P. pseudoalcaligenes* CECT5344 degrades jewelry industry wastewaters containing cyanide and metals in a batch reactor operating at an alkaline pH, but the awfully high tolerance of this strain to cyanate also allows improvement of the biodegradation process when cyanide is oxidized to cyanate with a pre-treatment of the residue with hydrogen peroxide.

## Figures and Tables

**Table 1:** Cyanase and nitrate reductase activities in the wild-type and CynF^−^ mutant strains of *P. pseudoalcaligenes* CECT5344 grown with different nitrogen sources.

Nitrogen Sources	Wild-Type Strain		CynF^−^ Mutant	
	Cyanase	Nitrate Reductase	Cyanase	Nitrate Reductase
-N	176 ± 21	114 ± 23	ND	201 ± 28
Ammonium	ND	13 ± 2	ND	235 ± 35
Nitrate	145 ± 18	202 ± 32	ND	283 ± 30
Glutamate	208 ± 27	86 ± 8	ND	241 ± 27
Cyanate	2677 ± 208	124 ± 15	ND	272 ± 31
Cyanate + ammonium	ND	19 ± 5	ND	242 ± 33
Cyanate + nitrate	2210 ± 115	194 ± 28	ND	290 ± 35
Cyanate + glutamate	2558 ± 223	97 ± 9	ND	243 ± 22

Cells were cultured with the indicated nitrogen sources (2 mM each) for 8 h, and enzyme activities were assayed as described in Materials and Methods, and expressed as mU/mg protein. Data are averages of three independent determinations ± standard deviation. -N, without added nitrogen source; ND, not detected.

## Supplementary Materials

Supplementary materials can be found at https://www.mdpi.com/1422-0067/20/12/3008/s1.
