# Supplementary material for: Cyanate Assimilation by the Alkaliphilic Cyanide-Degrading Bacterium Pseudomonas pseudoalcaligenes CECT5344: Mutational Analysis of the cyn Gene Cluster
Source: Int J Mol Sci. 2019 Jun 20;20(12):3008. doi: 10.3390/ijms20123008 (PMC6627978; doi:10.3390/ijms20123008)
Supplement: Supplementary file 1 [file ijms-20-03008-s001.pdf]

Supplementary Materials

Table S1. Bacterial strains and plasmids used in this study.

Strain or plasmid	Genotype and description[a]	Source/reference
***Pseudomonas pseudoalcaligenes* CECT5344**		
Wild-type	Nx^R	[1]
$CynS^-$	*cynS* mutant; Gm^R	[2]
$CynF^-$	*cynF* mutant; Km^R	This work
$CynBD^-$	*cynBD* mutant; Gm^R	This work
$CynX^-$	*cynX* mutant; Km^R	This work
$CynX^-/CynBD^-$	*cynX* and *cynBD* double mutant; Km^R and Gm^R	This work
Escherichia coli		
DH5α	*lac*$^-$, host for plasmids carrying *lacZ*	[3]
S17-1	*tra*$^+$, host for the mobilizable mob plasmids	[4]
**Plasmids**		
pBluescript KS (+)	Ap^R, cloning vector	Stratagene
pBmod	Ap^R, pBluescript Δ*Sal*I (deleted between *Eco*RV and *Hin*dII sites)	This work
pGEM-T Easy	Ap^R, cloning vector	Promega
pK18mob	Km^R, mobilizable suicide vector in *P. pseudoalcaligenes*, derived from pK18	[5]
pGEMT-*cynF*	pGEM-T Easy containing *cyn*F	This work
pBmod-*cynF*	pBmod containing *cynF*	This work
pBmod-*cynF::Km*	pBmod with *cynF::Km* insertion	This work
pK18mob-*cynF::Km*	pK18mob with *cynF::Km* insertion	This work
pGEMT-*cynB*	pGEM-T Easy containing *cynB*	This work
pK18mob-*cynD*	pK18mob containing *cynD*	This work
pK18mob-*cynBD*	pK18mob containing *cynB* and *cynD*	This work
pK18mob-*cynBD::Gm*	pK18mob with *cynBD::Gm* insertion	This work
pK18mob-*cynX*	pK18mob containing *cynX*	This work

[a]Abbreviations: Ap^R, ampicillin resistant; Gm^R, gentamicin resistant; Km^R, kanamycin resistant; Nx^R, nalidixic acid resistant.

Table S2. Oligonucleotides used in this work.

Primer	Sequence (5'→ 3')[a]	Used for
CynLF10	TCTCTGGGCGCATGACCTTGGGGCAC	*cynF* mutagenesis
CynLR7	CGGCTCGCGCAAGGAAGTGAAGAACG	*cynF* mutagenesis
CynLR9	TCTCCGCGCTGCTGTTGAAGCTTCTA (*Hin*dIII)	*cynBD* mutagenesis
CynLF11	CAACGTGGATCCAGTCGCGGCGCAC (*Bam*HI)	*cynBD* mutagenesis
CynLR8	CGGATCCGGCAAGTCGACCATTCTCA (*Bam*HI)	*cynBD* mutagenesis
CynLF8	CGGGCCGTTGGTCATCAGCAGAATTC (*Eco*RI)	*cynBD* mutagenesis
3113FB	CGGGATCCGGCAGTGCCATCGCGCTGGGTGCTGCCA (*Bam*HI)	*cynX* mutagenesis
3113RH	CGAAGCTTGCAGGTGACCAGCCCGCACGCCAGCAACA (*Hin*dIII)	*cynX* mutagenesis
Cyn1R	ATGCAGGATGCGGTCGTGGATTGGAT	RT-PCR
Cyn1F	TGGGAAAGCTCGCTGTCGGGGCTGAA	RT-PCR
Cyn2R	CGATCTCGAGGGGCAACTACCTCAACCAGCCGGTGCC	RT-PCR
Cyn2F	CCATCACGCTCAACGACTTGTAGGCATCCGCCGGTA	RT-PCR
CynBR	GGATTCGCAGACCTCGGCAATCTTCGT	RT-PCR
CynDF	CGGGAAGCGTTTGGCCAGGCGTCGG	RT-PCR
Cyn3R	CGATCCTGCTTTCCGACCGCATTCTGCTGATGACCAACG	RT-PCR
Cyn3F	TAGAAGAGGTAGATCAACGGGTCGGTGGGCACGGCCT	RT-PCR
rpoBF	AGCTGCTGCGTGCGATCTTCGGTGAGA	RT-PCR (housekeeping)
rpoBR	CCAATTGCTCGTTCAGGGCGTCGTCAG	RT-PCR (housekeeping)
qCynSF	TGCTCGAATTGCCGGCGGAGGTCTC	qRT-PCR
qCynSR	GGGTCGGTGGGCACGGCCTTG	qRT-PCR

[a]The sites for the restriction enzymes indicated in brackets are underlined.

```
                   Primer Cyn1R
GGCCGGATTGGCATGCAGGATGCGGTCGTGGATTGGATCGATGACCAGGCTGGCGCTCTGCATATGTTGCAGCAAC
CCGGCCTAACCGTACGTCCTACGCCAGCACCTAACCTAGCTACTGGTCCGACCGCGAGACGTATACAACGTCGTTG
 A  P  N  A  H  L  I  R  D  H  I  P  D  I  V  L  S  A  S  Q  M  H  Q  L  L

AACTGGCTGGCGGCATTGGGATCGGGCATTGCGGCGCTCTCGGGATTGACCTTTCAGGACGTTATCCAAAGGGCGT
TTGACCGACCGCCGTAACCCTAGCCCGTAACGCCGCGAGAGCCCTAACTGGAAAGTCCTGCAATAGGTTTCCCGCA
L  Q  S  A  A  N  P  D  P  M          S/D
                        CynF

GCCAGTTGACGATATCTCGTTTTTCAAGATGACGAAATCTCGTGAATAACGAGATTTCGTATTTATTGGTTTTGTT
CGGTCAACTGCTATAGAGCAAAAAGTTCTACTGCTTTAGAGCACTTATTGCTCTAAAGCATAAATAACCAAAACAA
                                                              -10

                                            σ54 (RpoN/NtrA)
AAAAGTTTATATTTTTCAATCGCTTATATAGATTTTTGAACTGGTACAGCTCCTGCAATAGCTGACATGAACCGGT
TTTTCAAATATAAAAAGTTAGCGAATATATCTAAAAACTTGACCATGTCGAGGACGTTATCGACTGTACTTGGCCA
              -35

                                                                     CynA
                                                         S/D          M  S
CAGCAATGGCCCAGCCCAGTCAGCGCCCGCCTCACCAATTTGGGGCAGGCCATCGCAGGAGTGATCACGATGAGCG
GTCGTTACCGGGTCGGGTCAGTCGCGGGCGGAGTGGTTAAACCCCGTCCGGTAGCGTCCTCACTAGTGCTAGTCGC

V  N  S  L  D  D  P  F  S  P  D  S  E  L  S  H  A  A  G  C  A  C  Q  R  C  T
TCAACAGCTTGGACGATCCATTCAGCCCCGACAGCGAGCTTTCCCATGCCGCCGGGTGTGCTTGCCAGCGTTGCAC
AGTTGTCGAACCTGCTAGGTAAGTCGGGGCTGTCGCTCGAAAGGGTACGGCGGCCCACACGAACGGTCGCAACGTG
                         Primer Cyn1F
```

Figure S1. Promoter region of the *cynF* and *cynA* genes of *P. pseudoalcaligenes* CECT5344. The *cynF* and *cynA* genes are divergently transcribed. The ATG start codons for both CynF and CynA proteins are indicated in red color, and their putative Shine-Dalgarno (S/D) sequences for ribosomal binding are shown in bold and underlined. The N-terminal amino acid sequences of CynF and CynA are also presented, with the corresponding DNA coding sequence shaded in grey. The putative σ^{70}-dependent promoter of the *cynF* gene (TATA box at position -10 and its upstream -35 sequence), and the σ^{54}-dependent promoter of the *cynA* gene (RpoN/NtrA binding site) are indicated. Primers Cyn1R and Cyn1F used for the amplification of the intergenic region are also marked.

References

1. Luque-Almagro, V.M.; Huertas, M.J.; Martínez-Luque, M.; Moreno-Vivián, C.; Roldán, M.D.; García-Gil, J.; Castillo, F.; Blasco, R. Bacterial degradation of cyanide and its metal complexes under alkaline conditions. *Appl. Environ. Microbiol.* **2005**, *71*, 940–947.
2. Luque-Almagro, V.M.; Huertas, M.J.; Sáez, L.P.; Martínez-Luque, M.; Moreno-Vivián, C.; Castillo, F.; Roldán, M.D.; Blasco, R. Characterization of the *Pseudomonas pseudoalcaligenes* CECT5344 cyanase, an enzyme that is not essential for cyanide assimilation. *Appl. Environ. Microbiol.* **2008**, *74*, 6280–6288.
3. Sambrook, J.; Russel, D.W. *Molecular Cloning: A Laboratory Manual*; Cold Spring Harbor Laboratory Press: Cold Spring Harbor, NY, USA, 2001.
4. Simon, R.; Priefer, U.; Pühler, A. A broad host range mobilization system for in vivo genetic engineering: Transposon mutagenesis in Gram-negative bacteria. *Biotechnology* **1983**, *1*, 784–791.
5. Schäfer, A.; Tauch, A.; Jäger, W.; Kalinowski, J.; Thierbach, G.; Pühler, A. Small mobilizable multi-purpose cloning vectors derived from the *Escherichia coli* plasmids pK18 and pK19: Selection of defined deletions in the chromosome of *Corynebacterium glutamicum*. *Gene* **1994**, *145*, 69–73.
